# Autoimmune Thyroid Diseases in Chronic Spontaneous Urticaria: The Role of Hormones, Anti-Thyroid Antibodies, and Ultrasound

**DOI:** 10.3390/diagnostics15050608

**Published:** 2025-03-03

**Authors:** Zoran Golušin, Nemanja Maletin, Nikola Denda, Miloš Nišavić, Bojan Radovanović, Olivera Nikolić

**Affiliations:** 1 Faculty of Medicine, University of Novi Sad, 21102 Novi Sad, Serbia; zoran.golusin@mf.uns.ac.rs (Z.G.); 902018d24@mf.uns.ac.rs (N.D.); milos.nisavic@mf.uns.ac.rs (M.N.); radovanovic.bojan@onk.ns.ac.rs (B.R.); olivera.nikolic@mf.uns.ac.rs (O.N.); 2 Clinic for Dermatovenerology, University Clinical Center of Vojvodina, 21102 Novi Sad, Serbia; 3 Department for Pathoanatomical Diagnostics, Department for Molecular Pathology, Oncology Institute of Vojvodina, 21208 Sremska Kamenica, Serbia; 4 Center for Radiology, University Clinical Center of Vojvodina, 21102 Novi Sad, Serbia

**Keywords:** chronic spontaneous urticaria, thyroid autoimmunity, anti-TPO antibodies, anti-TG antibodies, thyroid hormones, ultrasonography

## Abstract

**Background/Objectives:** Chronic spontaneous urticaria (CSU) is an immune-mediated skin disorder, with increasing evidence suggesting its association with autoimmune thyroid diseases. The presence of antithyroid antibodies (anti-TPO and anti-TG) and autoimmune thyroid disease indicates shared immunological mechanisms in the pathogenesis of both conditions. This study examines the prevalence of autoimmune thyroid changes in patients with CSU. **Methods:** The study was conducted as a combined retrospective-prospective observational analysis. It included 43 patients with CSU and 50 healthy participants in the control group. Thyroid hormone levels (TSH, T3, T4), anti-TPO and anti-TG antibodies, as well as ultrasound characteristics of the thyroid gland, were analyzed. **Results:** In patients with CSU, a higher prevalence of hypothyroidism (27.9% vs. 4% in the control group), hypertension, asthma, and diabetes were observed. Elevated levels of anti-TPO antibodies were found in 51.2% of CSU patients, compared to only 6% in the control group (*p* < 0.001). Similarly, anti-TG antibodies were increased in 41.9% of CSU patients, compared to 4% in the control group. Additionally, ultrasound analysis revealed significant differences in thyroid morphology, with a heterogeneous structure observed in 72.1% of CSU patients, compared to only 14% in the control group (*p* < 0.001). Nodular changes were present in 34.88% of CSU patients, whereas the prevalence in the control group was only 6% (*p* < 0.001). **Conclusions:** Our results confirm a significant association between CSU and autoimmune thyroid diseases, including a high prevalence of anti-TPO and anti-TG antibodies, hypothyroidism, diffuse heterogeneity, and nodular changes. Additionally, elevated T3 hormone levels were common among CSU patients, while T4 levels did not differ significantly from those in the control group.

## 1. Introduction

Chronic spontaneous urticaria (CSU) is a condition characterized by the spontaneous appearance of wheals (hives), angioedema, or both, lasting for more than six weeks and occurring and disappearing without an apparent cause [1]. The prevalence of CSU in the global population ranges between 0.02% and 5%, making it a significant clinical challenge [2]. The pathogenesis of CSU is not fully understood, but numerous studies suggest a significant role of autoimmune mechanisms, including autoimmune processes affecting the thyroid gland [3,4,5].

Recent findings have identified two main autoimmune mechanisms in CSU pathogenesis: type I autoallergic (associated with IgE antibodies against autoantigens) and type IIb autoimmune (driven by IgG autoantibodies targeting FcεRI or IgE) [6]. Distinguishing these mechanisms is clinically important, as they can influence disease severity and therapeutic response. The etiological factors of CSU include infections such as Helicobacter pylori and Candida, drug hypersensitivity, food allergies, and psychological stress [7]. Additionally, CSU frequently coexists with comorbidities such as autoimmune thyroid diseases, asthma, and metabolic disorders, further highlighting its complex and multifactorial nature [7].

For years, CSU was considered idiopathic, but the discovery of autoantibodies in certain patients led to the development of the concept of “autoimmune urticaria” [8]. Since urticaria and autoimmune thyroiditis share similar mechanisms of immune dysregulation, it is believed that these two diseases can often coexist in the same patient. The association between CSU and thyroid autoimmunity has been known since 1983 [9,10]. Reported prevalence varies across studies, indicating that the prevalence of thyroid autoantibodies or autoimmunity in patients with chronic urticaria ranges from 4.3% to 57%, while 5–10% of patients have clinically manifest thyroid disease [3,9].

Biomarkers play a crucial role in understanding the pathophysiology, diagnosis, and treatment of CSU, as they enable a more accurate assessment of disease activity, prediction of its course, and adjustment of therapeutic approaches. Standard biomarkers include total IgE, anti-TPO, and anti-TG antibodies, as well as thyroid function tests, which are particularly important due to the well-known association of CSU with autoimmune thyroid diseases, such as autoimmune thyroid disease [11]. Their presence may indicate prolonged disease duration and more severe clinical manifestations. Non-standard biomarkers, such as D-dimer, C-reactive protein (CRP), interleukin-6 (IL-6), matrix metalloproteinase-9 (MMP-9), and vascular endothelial growth factor (VEGF), are associated with disease severity, inflammatory responses, and coagulation abnormalities, which may suggest a poorer response to standard therapies [12].

Among many other biomarkers, one of the key indicators of autoimmune activity is the presence of thyroid peroxidase antibodies (anti-TPO), which are associated with thyroid diseases such as autoimmune thyroid disease [13,14]. In patients with CSU, the increased prevalence of elevated anti-TPO antibodies compared to the general population suggests a potential link between thyroid dysfunction and the development of urticaria [15]. Beyond laboratory findings, thyroid ultrasound frequently reveals structural abnormalities, including a heterogeneous gland texture, increased volume, or the presence of nodules, even in patients with hormone levels within the reference range [9]. Furthermore, thyroid autoimmunity is strongly associated with CSU, and findings from Asero et al. indicate that the presence of antithyroid antibodies may serve as a potential predictor of response to omalizumab therapy [16].

Although the autoimmune component of CSU has been widely studied, there are still a limited number of studies specifically analyzing the association between thyroid ultrasound findings, hormone levels, and the presence of anti-TPO antibodies in these patients. The aim of this study was to examine the frequency of these abnormalities in CSU patients and their prevalence compared to a control group, thereby contributing to a better understanding of the potential link between thyroid function and CSU.

## 2. Materials and Methods

### 2.1. Study Design

This study was designed as a combined retrospective-prospective observational analysis. The retrospective part includes an analysis of medical records of patients diagnosed with CSU who were treated at a tertiary healthcare center over the past three years. These data include laboratory findings of thyroid hormones (T3, T4, and TSH), the presence of anti-TG and anti-TPO antibodies, and thyroid ultrasound examinations. The prospective part of the study involves active monitoring of new patients diagnosed with CSU over 12 months, during which additional laboratory tests and ultrasound examinations were conducted to supplement the data and assess longitudinal changes. The study commenced on 1 January 2022 and concluded on 31 December 2024.

The research was conducted at the University Clinical Center of Vojvodina (UCCV), at the Clinic for Dermatovenereology and the Radiology Center. Dermatologists from the Clinic for Dermatovenereology were responsible for patient recruitment and symptom severity assessment, while radiologists from the Radiology Center at UCCV performed standardized thyroid ultrasound examinations. Laboratory diagnostics, including the analysis of thyroid hormones (T3, T4, and TSH) and anti-Tg and anti-TPO antibodies, were carried out at the UCCV Laboratory Diagnostics Center using validated immunological and biochemical testing methods. A multidisciplinary team ensured high-quality diagnostics and data accuracy, allowing for a detailed analysis of the relationship between CSU and thyroid function.

### 2.2. Participants

The study participants were divided into two groups. The first group consisted of 43 patients diagnosed with CSU, while the control group included 50 healthy individuals with similar demographic characteristics. Inclusion in the study required meeting specific criteria, such as being at least 18 years old and having a confirmed CSU diagnosis according to the international EAACI/GA^2^LEN/WAO guidelines [1]. In the study group, patients with autoimmune diseases other than autoimmune thyroid disease were excluded, as well as those who had used immunosuppressive drugs in the past six months. All participants in the control group were healthy individuals based on medical history data, with no CSU diagnosis and no other autoimmune diseases.

### 2.3. Methods

In the study, data were collected using a survey questionnaire, which included demographic, clinical, and other relevant information related to the respondents. Demographic data included information on gender, age (expressed as average age and age categories), and place of residence (urban or rural). Clinical data encompassed the duration of the disease, the current status of disease episodes (whether changes were present or not), the frequency of symptoms throughout the year, the duration of changes expressed in hours, as well as the time of day when symptoms were most pronounced (during the day, at night, or with no specific difference). The Urticaria Activity Score over seven days (UAS7) was used to assess disease severity. Additionally, data were collected on specific situations in which symptoms were more pronounced, such as travel, vacations, or weekends. The survey questions related to health status included the presence of allergies, various infections (*H. pylori*, *Candida*, *E. coli*), as well as associated diseases (comorbidities), including hypothyroidism, hypertension, asthma, diabetes, depression, and other disorders.

Thyroid hormones, including TSH (thyroid-stimulating hormone), T3 (triiodothyronine), and T4 (thyroxine), were analyzed using immunological methods such as chemiluminescence, while anti-Tg antibodies (anti-thyroglobulin antibodies) and anti-TPO antibodies (anti-thyroid peroxidase antibodies) were measured using ELISA tests. The reference values used for analysis were as follows: TSH: 0.4–4.0 mIU/L, T3: 0.8–2.0 ng/mL, and T4: 57.9–161 nmol/L. Elevated levels of anti-Tg (>35 IU/mL) and anti-TPO antibodies (>35 IU/mL) were considered indicators of autoimmune processes in the thyroid gland. Analyses were performed on Cobas e411 analyzers (Roche Diagnostics, Basel, Switzerland) for TSH, T3, and T4 using chemiluminescent methods, while anti-Tg and anti-TPO antibodies were measured using ELISA tests on an ELx808 device (BioTek Instruments, Vermont, USA). All samples were analyzed immediately according to standard protocols to minimize errors and ensure result reliability. Blood samples were collected via venipuncture in the morning after at least eight hours of fasting to ensure result accuracy.

During the ultrasound examination of the thyroid gland, the size of the thyroid (expressed in cubic centimeters), its structure (categorized as homogeneous or heterogeneous), and the presence of nodules and cysts were assessed. The ultrasound examinations were performed with the patient in a supine position with the head slightly tilted back, allowing for an optimal view of the thyroid gland. The examinations were performed using high-frequency range devices (7.5–15 MHz) on a GE LOGIQ P7 ultrasound machine.

### 2.4. Statistical Analysis

Data analysis was performed using SPSS software (IBM SPSS Statistics, version 27.0.2). Descriptive statistics were used to process the data. Comparisons between groups (CSU patients and the control group) were conducted using the chi-square test for categorical variables. Statistical significance was set at *p* < 0.05. The results were visualized in tables and graphs for clear comparisons between groups.

### 2.5. Ethical Considerations

All study procedures adhered to the Declaration of Helsinki [17], and the study protocol was approved by the Ethics Committee of UCCV (approval decision dated 31 January 2025, approval number: 00-58). Participation in this study was voluntary, anonymous, and without financial compensation. Each participant provided informed consent before inclusion in the study. A unique identification number was assigned to each participant, after which all data were entered into a secured database with restricted access available only to research team members. All analysis results were presented in an aggregated form, ensuring the complete anonymity of each participant.

## 3. Results

The study included 43 patients with CSU and 50 participants in the control group (Table 1). Most participants in both groups were female, with 88.4% of patients in the CSU group being women. The average age of participants was similar, 47.2 years in the CSU group and 46.3 years in the control group. The detailed demographic data are presented in Table 1.

Regarding infections, the most commonly identified infections in the CSU group were caused by Helicobacter pylori (27.3%), followed by Candida (22.7%). In the CSU group, 46.5% of patients reported having allergies, while the remaining 53.5% stated they had no allergies. Patients with CSU had a significantly higher prevalence of comorbidities, including hypothyroidism (27.9% compared to 4% in the control group) and hypertension (19.7% compared to 2%). Additionally, asthma and diabetes were recorded exclusively in the CSU group, while the majority of participants in the control group (92%) had no comorbidities (*p* < 0.001). Symptoms in CSU patients were often more pronounced at night (44.2%) and during weekends (89.7%). Furthermore, skin changes occurred less than three times per year in most patients (69.8%). The detailed clinical data are shown in Table 2.

**Figure 1:**
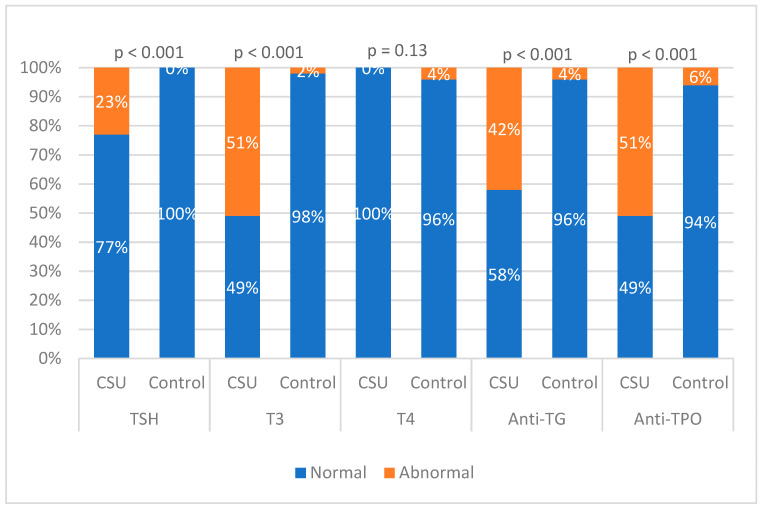
Percentage of patients with elevated thyroid antibodies and hormone levels (CSU vs. Control).

The analysis of thyroid function and antibody levels revealed significant differences between patients with CSU and the control group (Table 3). Elevated TSH levels were observed in 23.3% of CSU patients, while no cases were reported in the control group (*p* < 0.001). Similarly, high T3 levels were present in 51.2% of CSU patients compared to only 2% in the control group (*p* < 0.001). Thyroid autoantibodies showed a marked difference, with 41.9% of CSU patients having elevated anti-TG levels and 51.2% with elevated anti-TPO levels, in contrast to only 4% and 6%, respectively, in the control group (*p* < 0.001). Additionally, no significant difference was found in T4 levels between the groups (*p* = 0.13). The most pronounced differences were observed for anti-TPO and T3, where the proportion of CSU patients with abnormal values was significantly higher than in the control group. The graphical representation of these results is shown in Figure 1.

The evaluation of thyroid characteristics showed notable differences between patients with CSU and the control group (Table 4). Thyroid size was predominantly normal in both groups, but 11.63% of CSU patients had an enlarged thyroid compared to 4% in the control group. Additionally, thyroid nodules were significantly more frequent in the CSU group (34.88%) than in the control group (6%) (*p* < 0.001). Thyroid structure also differed, with 72.1% of CSU patients showing heterogeneous thyroids, compared to only 14% in the control group (*p* < 0.001). Autoimmune thyroid disease was diagnosed in 32.55% of CSU patients, contrasting sharply with 5% in the control group (*p* < 0.001).

## 4. Discussion

The main findings of the study indicate a significant association between CSU and autoimmune processes in the thyroid gland. Elevated anti-TPO antibody levels were recorded in 51.2% of patients with CSU, compared to only 6% in the control group (*p* < 0.001). Similarly, anti-TG antibodies were elevated in 41.9% of CSU patients compared to 4% in the control group. Additionally, ultrasound analysis revealed a significant difference in thyroid morphology, with a heterogeneous structure observed in 72.1% of CSU patients, compared to only 14% in the control group (*p* < 0.001). Nodular changes were present in 34.88% of CSU patients, whereas only 6% of the control group exhibited such changes (*p* < 0.001). These results strongly suggest a link between autoimmune thyroid alterations and CSU pathogenesis, highlighting the need for further research into this association.

Autoimmune thyroid disease has a prevalence of 2% in the general population [18]. Autoimmune thyroid disease is an autoimmune disease characterized by thyroid inflammation, follicular destruction, and consequent hypothyroidism, with thyroid hormone replacement being the primary treatment [19]. The etiology of autoimmune thyroid disease is multifactorial, involving genetic predisposition, environmental exposures, and specific genetic polymorphisms [20]. Chiu et al. identified significant associations between chronic urticaria, atopic and autoimmune diseases (asthma, atopic dermatitis, allergic rhinitis, systemic lupus erythematosus, autoimmune thyroid diseases, psoriasis, Kawasaki disease, Sjögren’s syndrome, Henoch–Schönlein purpura, and inflammatory bowel diseases), highlighting shared genetic components and etiological pathways [21]. The pathogenesis of CSU may be linked to thyroid autoimmune processes, with autoantibodies and inflammatory cytokines playing a key role. The study by Carlucci et al. examines the shared immunological pathways between autoimmune thyroid and skin diseases, further supporting the hypothesis of a potential immunological overlap between CSU and thyroid disorders [22].

It has been reported that 42% of patients with chronic urticaria exhibit elevated TSH levels, even in the absence of clinical hypothyroidism [23]. TSH receptors are present not only in the thyroid gland, but also in immune system cells. Moreover, TSH itself can act as a cytokine in thyroid diseases and is associated with the continuous release of IL-1, IL-2, IL-6, and IL-12 by lymphocytes and dendritic cells, further enhancing immune responses and mast cell activation [23]. In our study, 23% of patients with CSU had elevated TSH levels. Although the mechanisms underlying the connection between CSU and thyroid autoimmunity remain unclear, evidence supports an immunoregulatory role of TSH. Some studies suggest that levothyroxine therapy may improve urticaria symptoms [24]. However, these findings remain controversial, as other studies have failed to confirm this effect [13]. These discrepancies may result from small sample sizes, varying diagnostic criteria, and the presence of other immunological factors that influence CSU pathogenesis. Additionally, some authors have noted that CSU patients with anti-TPO antibodies are more likely to develop hypothyroidism or subclinical thyroid dysfunction, although there is no clear correlation between TSH levels and urticaria severity [23,25]. In our study, hypothyroidism was the most common comorbidity among CSU patients, affecting 28% of participants. In previous research, hypothyroidism prevalence in CSU patients ranged from 2% to 37% [26].

Our study identified CSU patients with low TSH levels and others with elevated T3 levels, indicating the presence of both hypothyroid and hyperthyroid patterns within the study population. We found a significant difference in T3 levels between CSU patients and the control group, with 51% of CSU patients exhibiting elevated T3 levels. In contrast, T4 levels did not show significant differences between the two groups. Similar results were reported in a study by Czarnecka-Operacz et al., where T3 levels were significantly higher in CSU patients, while T4 levels remained unchanged compared to the control group [3]. The clinical implications of these findings are particularly significant. Hypothyroidism, especially subclinical forms, has been associated with increased severity of CSU due to impaired immune tolerance and chronic inflammatory responses [8]. Elevated levels of anti-thyroperoxidase (anti-TPO) and anti-thyroglobulin (anti-Tg) antibodies frequently accompany hypothyroidism, suggesting an autoimmune basis that may contribute to persistent urticarial symptoms [3]. On the other hand, hyperthyroidism, although less common, presents specific clinical challenges. Elevated T3 levels in CSU patients may indicate a hypermetabolic state that influences mast cell degranulation, leading to increased histamine release and more severe urticarial manifestations [20]. Furthermore, hyperthyroidism is often associated with Graves’ disease, where thyroid-stimulating immunoglobulins can further disrupt immune balance, potentially exacerbating CSU symptoms [26].

Systematic reviews and meta-analyses have demonstrated that CSU patients have a significantly higher prevalence of anti-TPO antibodies compared to control groups, suggesting a potential immunological link between these conditions [8,26]. A meta-analysis covering 19 studies with a total of 14,351 CSU patients and 12,404 controls found that CSU patients were five to seven times more likely to test positive for anti-TPO antibodies (OR 5.18, 95% CI 3.27–8.22, *p* < 0.00001) [8]. Kohlir et al. reported that CSU patients frequently exhibit elevated IgG anti-thyroid autoantibodies, further supporting an autoimmune mechanism as a potential cause of the disease [26]. The association between anti-TPO antibodies and CSU can be explained by shared immune mechanisms, including T-regulatory cell (Tregs) dysfunction, increased IL-6 expression, Th17 lymphocyte activity, and potential “autoallergic” mechanisms via IgE antibodies against thyroid antigens [18,27,28]. The presence of anti-TPO antibodies signals an increased immune response against self-tissues, including mast cells and basophils, which play a key role in CSU pathogenesis [14]. Tregs dysfunction has been observed in both autoimmune thyroid diseases and CSU, allowing for uncontrolled autoimmune activity and increased autoantibody production [29,30]. Due to Tregs cell dysfunction, Th1 and Th2 cells are inhibited in autoimmune thyroid disease [27]. Studies have shown that some CSU patients have IgE antibodies targeting the TPO enzyme, potentially leading to mast cell activation and histamine release, similar to classical allergic reactions [20,31]. Additionally, the activation of the complement factors C3a and C5a can further stimulate mast cell degranulation, contributing to inflammatory processes and potentially worsening CSU symptoms in patients with autoimmune thyroid diseases [15,26]. In our study, elevated anti-TPO antibody levels were observed in 51.2% of CSU patients, compared to only 6% in the control group, highlighting the increased prevalence of thyroid autoimmunity in this population.

Numerous studies have demonstrated that CSU patients have a significantly higher prevalence of anti-Tg antibodies compared to healthy controls, further confirming the immune-mediated basis of this disease [8,32,33]. The presence of anti-Tg antibodies indicates the activation of autoreactive B-lymphocytes, which may contribute to an inflammatory environment and increased production of pro-inflammatory cytokines such as IL-6 and TNF-α [27]. These factors can influence mast cell activation, which plays a key role in CSU development. The presence of anti-Tg antibodies in CSU patients further supports the autoimmune component in disease pathogenesis. CSU patients with anti-TPO antibodies often exhibit other immune abnormalities, including elevated pro-inflammatory cytokines, complement system activation, and potential autoallergic mechanisms. Previous research has shown that anti-Tg antibody prevalence in CSU patients ranges from 16% to 42% [15,22]. In our study, 42% of CSU patients tested positive for anti-Tg antibodies, aligning with these findings and further confirming the association between thyroid autoimmunity and CSU pathogenesis. A study involving 148 CSU patients and 148 healthy controls found a significant difference in anti-Tg and anti-TPO antibody prevalence between the two groups [3]. Specifically, anti-TPO antibodies were significantly more common in CSU patients, with an odds ratio (OR) of 6.69 (*p* = 0.0045), while anti-Tg antibody prevalence was also significantly increased (OR 6.01, *p* = 0.013) [3].

Ultrasound examination of the thyroid gland in patients with CSU often reveals significant morphological changes suggestive of autoimmune processes in the thyroid. Studies have confirmed that CSU patients have an increased prevalence of heterogeneous thyroid structure on ultrasound findings, including hypoechogenicity, the presence of nodular changes, and reduced vascularization, which are characteristic of autoimmune thyroid diseases such as autoimmune thyroid disease [34]. In ultrasounds, autoimmune thyroid disease is manifested by hypoechogenicity, a heterogeneous structure, pseudonodules, increased vascularization, possible calcifications, and changes in gland size [34]. Thyroid hypoechogenicity is one of the most frequent ultrasound findings in patients with autoimmune thyroiditis. Studies have shown that CSU patients have a significantly higher prevalence of diffuse thyroid heterogeneity, which may indicate ongoing inflammation and lymphocytic infiltration [33]. In our study, diffuse thyroid heterogeneity was observed in 72% of CSU patients, whereas this finding was present in only 14% of control subjects. Nodular thyroid changes were detected in a significant proportion of CSU patients. In a study by Su et al., thyroid nodules were significantly more frequent in CSU patients (56% vs. 36% in the control group, *p* = 0.045) [35]. Additionally, papillary thyroid carcinoma was confirmed in 8% of CSU patients, whereas it was detected in only 2% of control subjects [35]. In our study, 35% of CSU patients had thyroid nodules, compared to only 6% in the control group. These findings suggest a potential association between CSU and an increased risk of nodular and malignant thyroid changes, emphasizing the need for careful monitoring of these patients. The presence of micronodules may indicate chronic inflammation and fibrotic changes, which are characteristic of autoimmune thyroid processes [34]. Ultrasound abnormalities in CSU patients are often associated with the presence of anti-TPO and anti-Tg antibodies. Additionally, some studies have shown that CSU patients with more pronounced thyroid ultrasound abnormalities tend to have more severe CSU symptoms and poorer responses to standard therapy [6]. The prevalence of autoimmune thyroid disease in previous studies ranged from 1% to 28%, while in our study, it was diagnosed in 33% of CSU patients [26].

Our study has certain limitations that should be considered. The relatively small sample size may restrict the generalizability of the results to a broader population, while selection bias, due to recruiting participants from a specific population, may further impact the applicability of the findings. The lack of longitudinal follow-up makes it difficult to assess long-term changes in thyroid function and their impact on the course of CSU. Although standardized methods were used for hormone and antibody measurements, potential variations in laboratory procedures could affect the accuracy of results and their comparability with other studies. Additionally, other factors such as coexisting autoimmune diseases and external influences were not analyzed, which could contribute to the development of CSU and autoimmune thyroid processes. Moreover, the lack of analysis of therapeutic responses in patients with positive antithyroid antibodies limits insights into the clinical implications of the findings. Future research with larger sample sizes, a longitudinal design, and a broader range of analyzed factors could contribute to a deeper understanding of the relationship between CSU and autoimmune thyroid diseases.

## 5. Conclusions

Our results indicate a significant association between CSU and autoimmune processes in the thyroid gland, confirming the high prevalence of anti-TPO and anti-TG antibodies, as well as morphological changes detected through ultrasound in patients with CSU. Patients with CSU had a significantly higher prevalence of hypothyroidism, diffuse thyroid heterogeneity, and nodular changes compared to the control group. Additionally, elevated T3 hormone levels were observed in a significant number of CSU patients compared to the control group, while T4 levels did not show a significant difference between the groups.

These findings highlight the need for routine thyroid function assessment in CSU patients, especially those with clinical signs or laboratory markers of autoimmune changes. Early recognition and treatment of thyroid dysfunction may contribute to better clinical outcomes and improved quality of life for patients. Further research is necessary to clarify the pathogenic link between CSU and autoimmune thyroid diseases more precisely, as well as the potential therapeutic implications of this connection.

## Figures and Tables

**Table 1:** Demographic characteristics of participants.

Variables	CSU (n = 43)	Control Group (n = 50)	*p*-Value
Gender, n (%)			0.57
Male	5 (11.6)	9 (18)	
Female	38 (88.4)	41 (82)	
Age, mean (SD)	47.2 (14.8)	46.30 (15.12)	
Age category, n (%)			0.78
18–39	15 (35.7)	15 (30.0)	
40–64	23 (54.8)	31 (62.0%)	
>65	4 (9.5)	4 (8.0%)	
Resident area, n (%)			
Urban	21 (48.8)	NA	
Rural	22 (51.2)		
Occupation, n (%)			
Homemaker	11 (25.6)	NA	
Retiree	7 (16.3)		
Economist	7 (16.3)		
Salesperson	4 (9.3)		
Other	14 (32.6)		
Marital Status, n (%)			
Married	27 (62.8)	NA	
Single	16 (37.2)		

**Table 2:** Clinical characteristics of participants.

Variables	CSU (n = 43)	Control Group (n = 50)	*p*-Value
Duration of Illness, n (%)			
<5 years	24 (55.8)	NA	
5–10 years	4 (9.3)		
>10 years	13 (30.2)		
Current Episode, n (%)			
Hives	22 (51.2)	NA	
Without hives	21 (48.8)		
Frequency of Occurrence Per Year, n (%)			
None	2 (4.7)	NA	
Low (1–3 times)	30 (69.8)		
Moderate (4–6 times)	6 (14.0)		
High (>6 times)	5 (11.6)		
Duration of Changes, n (%)			
<12 h	28 (65.1)	NA	
12–24 h	9 (20.9)		
24–72 h	6 (14.0)		
When Symptoms Are Worse, n (%)			
Day	11 (25.6)	NA	
Night	19 (44.2)		
No Difference	13 (30.2)		
Symptoms During Specific Times, n (%)			
During Travel	18 (62.1)	NA	
During Vacation	22 (75.9)		
During Weekends	26 (89.7)		
Allergies, n (%)			*p* < 0.001
Yes	20 (46.5)	8 (16)	
No	23 (53.5)	42 (84)	
Infections, n (%)			*p* < 0.001
*H. pylori*	12 (27.3)	2 (4)	
*Candida*	10 (22.7)	3 (6)	
*E. coli*	3 (6.8)	1 (2)	
None	19 (43.2)	44 (88)	
Comorbidities, n (%)			*p* < 0.001
Hypothyroidism	17 (27.9)	2 (4)	
Hypertension	12 (19.7)	1 (2)	
Asthma	4 (6.6)	0 (0)	
Diabetes mellitus	4 (6.6)	0 (0)	
Depression	3 (4.9)	0 (0)	
Other	10 (16.4)	1 (2)	
None	11 (18)	46 (92)	

**Table 3:** Thyroid function and antibody levels.

Variables	CSU (n = 43)	Control Group (n = 50)	*p*-Value
TSH 0.4–4.0 µIU/mL			
Normal	31 (72.1)	50 (100)	*p* < 0.001
High	10 (23.3)	0 (0)	
Low	2 (4.7)	0 (0)	
Mean (SD)	3.58 (3.76)	2.2 (1.07)	
Median (rang)	2.30 (0.05–16.70)	2.25 (0.42–3.95)	
T3 0.8–2.0 ng/mL			
Normal	21 (48.8)	47 (94)	*p* < 0.001
High	22 (51.2)	1 (2)	
Low	0 (0)	2 (4)	
Mean (SD)	2.82 (1.42)	1.16 (0.52)	
Median (rang)	2.2 (1.2–5.6)	1.10 (0.16–2.62)	
T4 57.9–161 nmol/L			
Normal	41 (95.3)	48 (96)	0.13
High	0 (0)	2 (4)	
Low	2 (4.7)	0 (0)	
Mean (SD)	89.53 (25.99)	134.07 (67.12)	
Median (rang)	84.00 (50.00–161.00)	123.63 (58.42–296.79)	
Anti TG ≤ 35 IU/mL			
Normal	25 (58.1)	48 (96)	*p* < 0.001
High	18 (41.9)	2 (4)	
Mean (SD)	211.18 (541.38)	20.97 (10.99)	
Median (rang)	20.00 (0.00–2943.00)	21.807 (0.541–44.115)	
Anti TPO ≤ 35 IU/mL			
Normal	21 (48.8)	47 (94)	*p* < 0.001
High	22 (51.2)	3 (6)	
Mean (SD)	288.38 (378.47)	19.279 (11.722)	
Median (rang)	40.00 (0.00–1000.00)	18.95 (1.06–47.28)	

**Table 4:** Thyroid morphology and structural characteristics on ultrasonography.

Variables	CSU (n = 43)	Control Group (n = 50)	*p*-Value
Size, n (%)			0.38
Normal	34 (79.07)	43 (86)	
Enlarged	5 (11.63)	2 (4)	
Atrophic	4 (9.3)	5 (10)	
Volume of the right lobe (cm^3^)			
Mean (SD)	9543.23 (5018.56)	NA	
Mediana (rang)	8370 (1440–24,000)	NA	
Volume of the left lobe (cm^3^)			
Mean (SD)	9131.47 (4576.35)	NA	
Mediana (rang)	8064 (1584–21,600)	NA	
Istmus			
Mean (SD)	2.76 (1.2)	NA	
Mediana	2.5	NA	
Cyst, n (%)			0.56
Yes	6 (13.9)	4 (8)	
No	37 (86.1)	46 (92)	
Nodule, n (%)			*p* < 0.001
Yes	15 (34.88)	3 (6)	
No	28 (65.12)	47 (94)	
Structure			*p* < 0.001
Homogeneous	12 (27.9)	43 (86)	
Heterogeneous	31 (72.1)	7 (14)	
Autoimmune thyroid disease			*p* < 0.001
Yes	14 (32.55)	2 (5)	
No	30 (67.45)	48 (95)	

## Data Availability

The original contributions presented in this study are included in the article. Further inquiries can be directed to the corresponding author(s).
